# Supplementary material for: Structures of BCL-2 in complex with venetoclax reveal the molecular basis of resistance mutations
Source: Nat Commun. 2019 Jun 3;10:2385. doi: 10.1038/s41467-019-10363-1 (PMC6547681; doi:10.1038/s41467-019-10363-1)
Supplement: Supplementary file 1 — Supplementary Information [file 41467_2019_10363_MOESM1_ESM.pdf]

**Supplementary Information**

# Structures of BCL-2 in complex with venetoclax reveal the molecular basis of resistance mutations

Richard W. Birkinshaw, *et al.*.

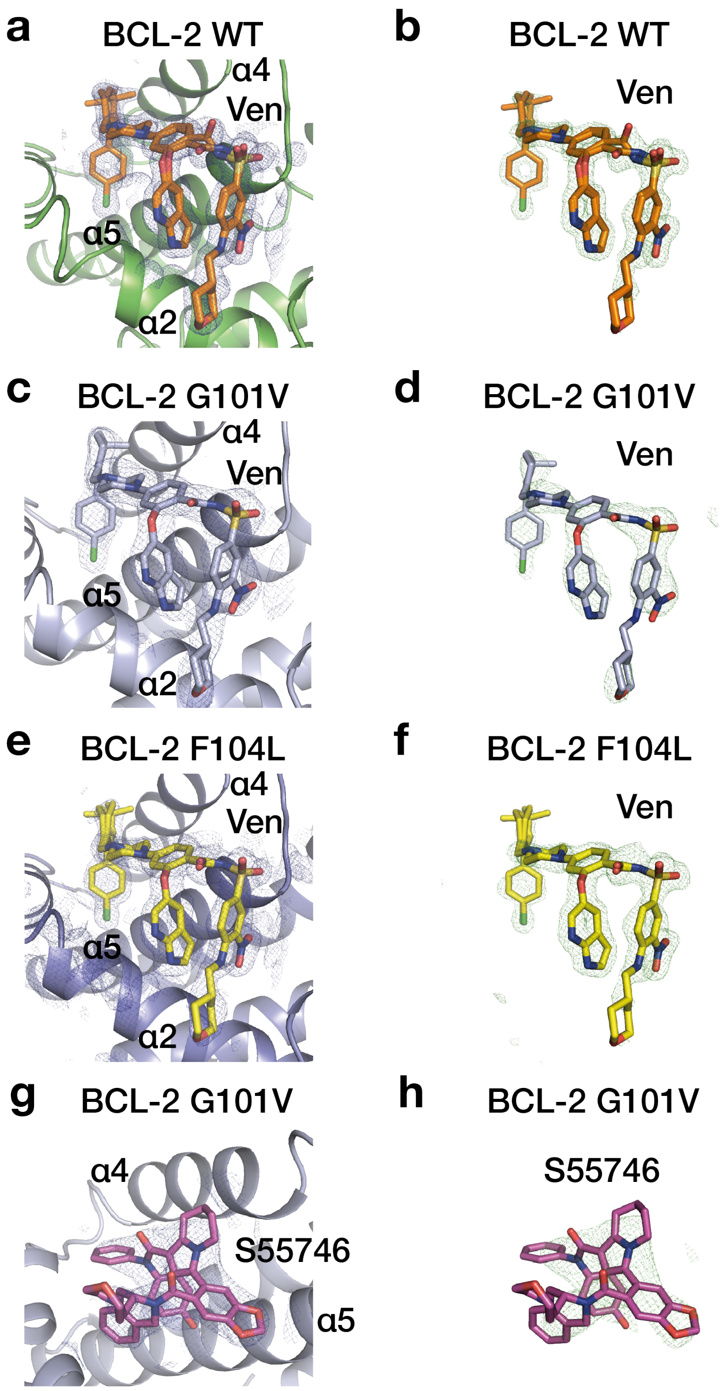


**Supplementary Figure 1. BCL-2 mutant with Venetoclax or S55746 electron density maps.** Electron density maps for BCL-2 mutants with venetoclax (Ven) or S55746 showing sigma weighted 2Fo-Fc refined density (blue; **a**, **c**, **e**, **g**) or simulated annealing (cartesian) Fo-Fc omit map density (green; **b**, **d**, **e**, **f)** for each crystal structure with BCL-2 mutants: wild-type (WT, green), G101V (light blue) and F104L (darker blue). The 2Fo-Fc density was contoured at 1.0 σ and Fo-Fc density contoured at 3.0 σ. Maps were visualised in pymol using the isomesh function, excluding density 3 Å from either venetoclax or S55746.

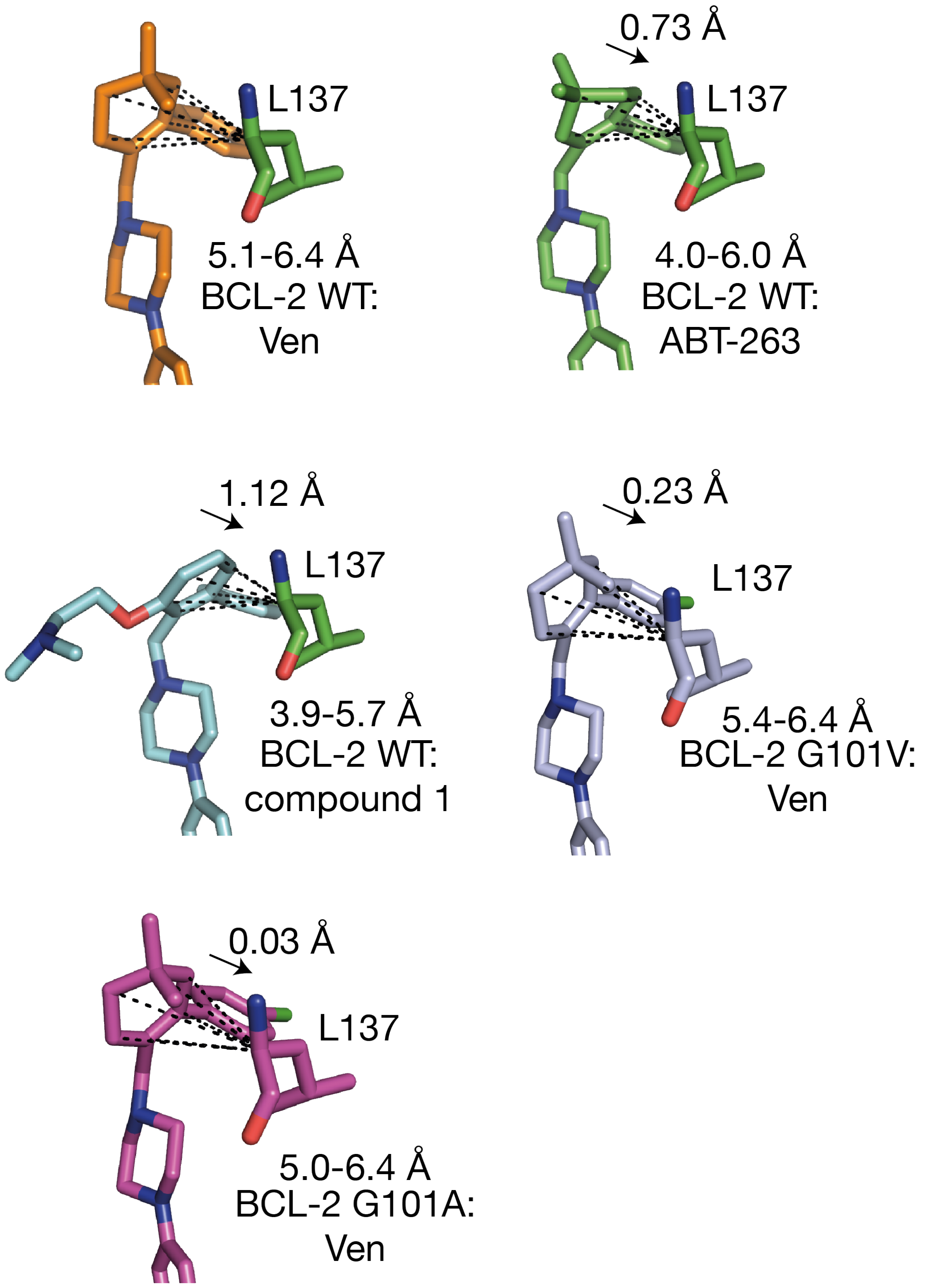


**Supplementary Figure 2. Relative distances between compounds and L137.** Distances were measured (black dotted lines) between the top 6 membered ring of venetoclax (Ven, orange or light blue), ABT-263 (green) and 4MAN analogue (cyan) to the BCL-2 residue L137 Cα. The range of these distances are shown for each structure. RMSD values relative to the BCL-2 WT:venetoclax (alternate conformer A) are indicated above arrows informing the movement of the ring towards L137.

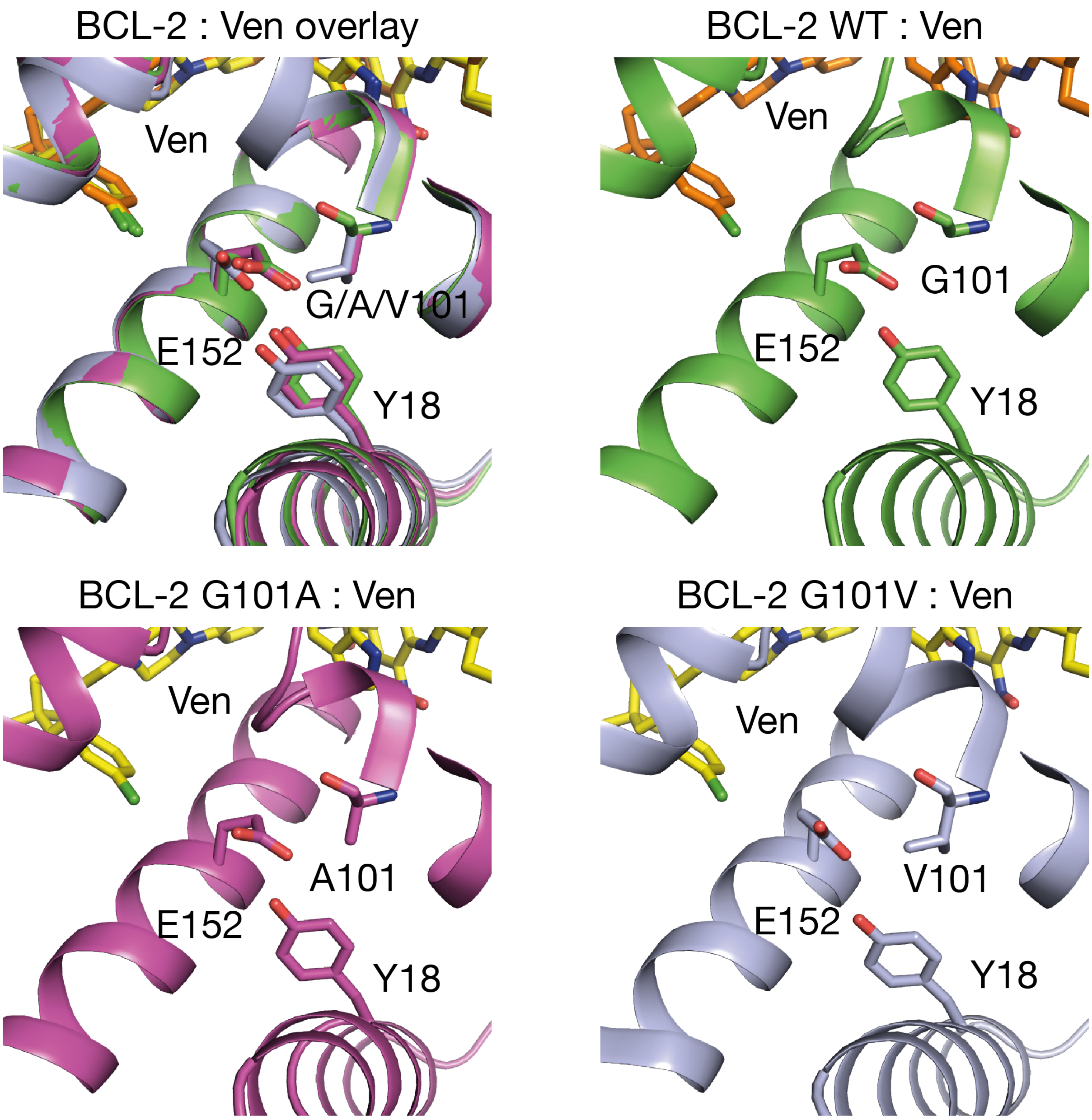


**Supplementary Figure 3. Structure of BCL-2 G101A:venetoclax.** Structures of BCL-2 WT (green), G101A (magenta) and G101V (light blue) bound to venetoclax (Ven, orange in WT and yellow in mutant structures). Key residues Tyr18, Glu152 and Gly/Ala/Val101 are shown in stick representation.

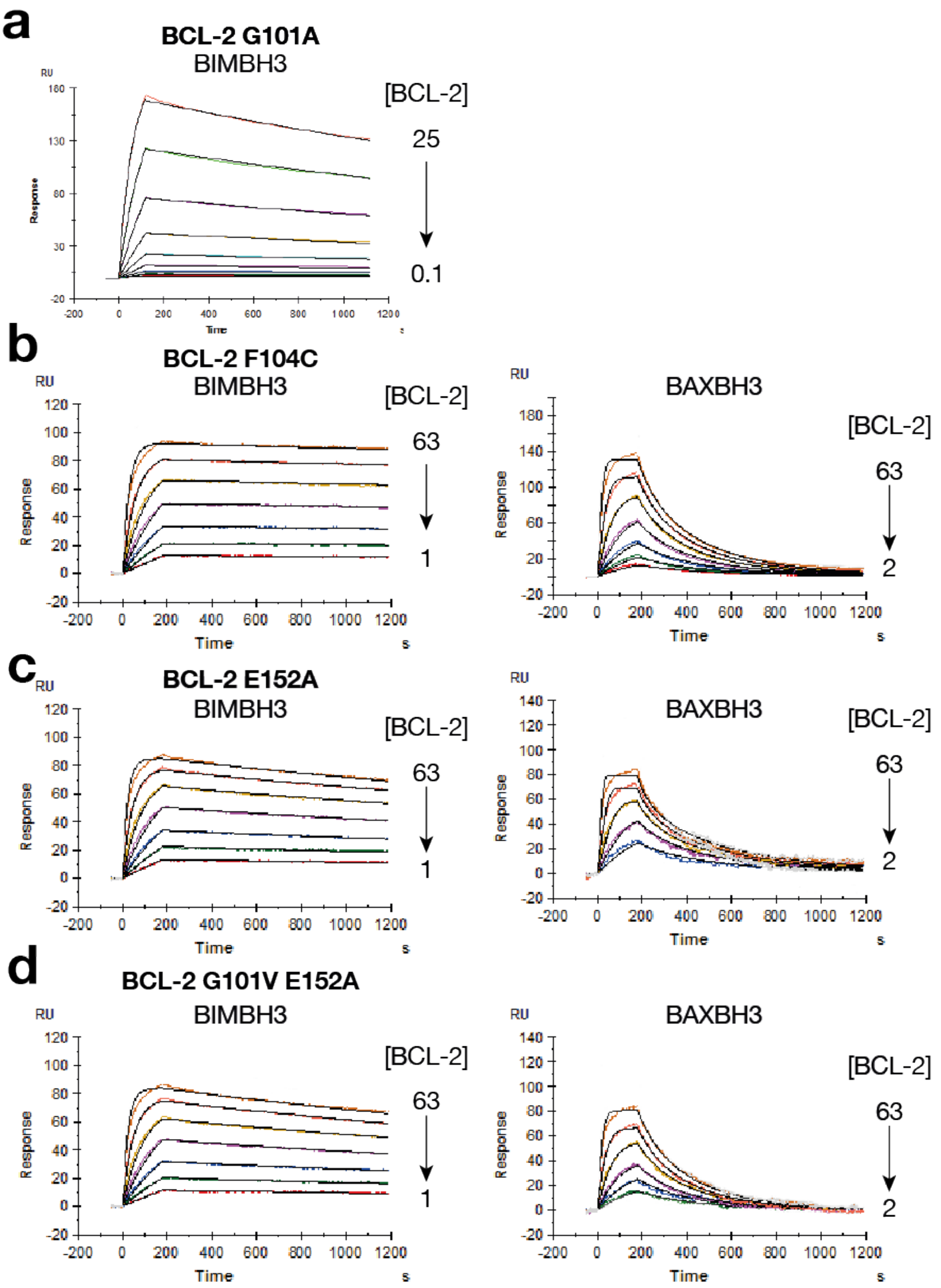


**Supplementary Figure 4. SPR sensograms of BCL-2 mutants binding to BIMBH3 and BAXBH3 peptides.** Direct binding experiments to determine affinity of BCL-2 mutants G101A (**a**), F104C (**b**), E152A (**c**), G101V E152A double mutant (**d**) for BIMBH3 and BAXBH3 peptides. Raw data (coloured curves) were fitted (black curves) to a 1 site specific kinetic model to determine on and off rates and the equilibrium binding constant $K_D$. Data are representative of at least 2 independent experiments. The BCL-2 mutant proteins were diluted in a 2-fold dilution series, concentration ranges for curves are indicated. All experiments shown were performed on a BIAcore 4000, with the exception of (**a**) which was performed on a BIAcore S200.

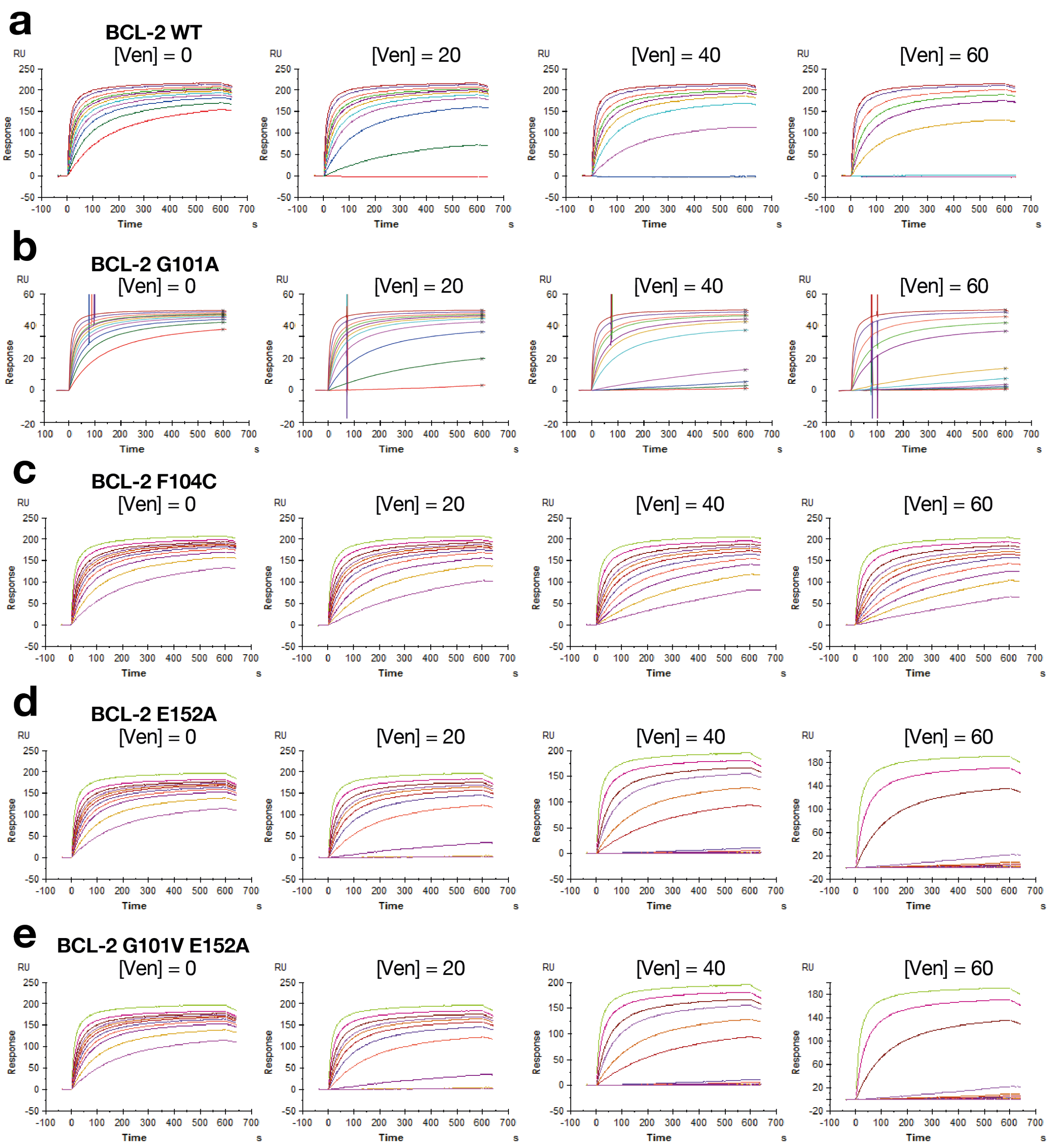


**Supplementary Figure 5. BCL-2 mutants with venetoclax steady-state SPR sensograms.** Double referenced sensorgrams for BCL-2 wild-type (WT, **a**), G101A (**b**), F104C (**c**), E152A (**d**) and G101V E152A (**e**) double mutant (**d**) binding to a BIMBH3 peptide immobilised chip in the presence of various venetoclax (Ven) concentrations (0-60 nM). Average response at the end of injection ~600 sec, were plotted as a function of BCL-2 and venetoclax concentration in figure 3a-d. Data are representative of 3 independent experiments.

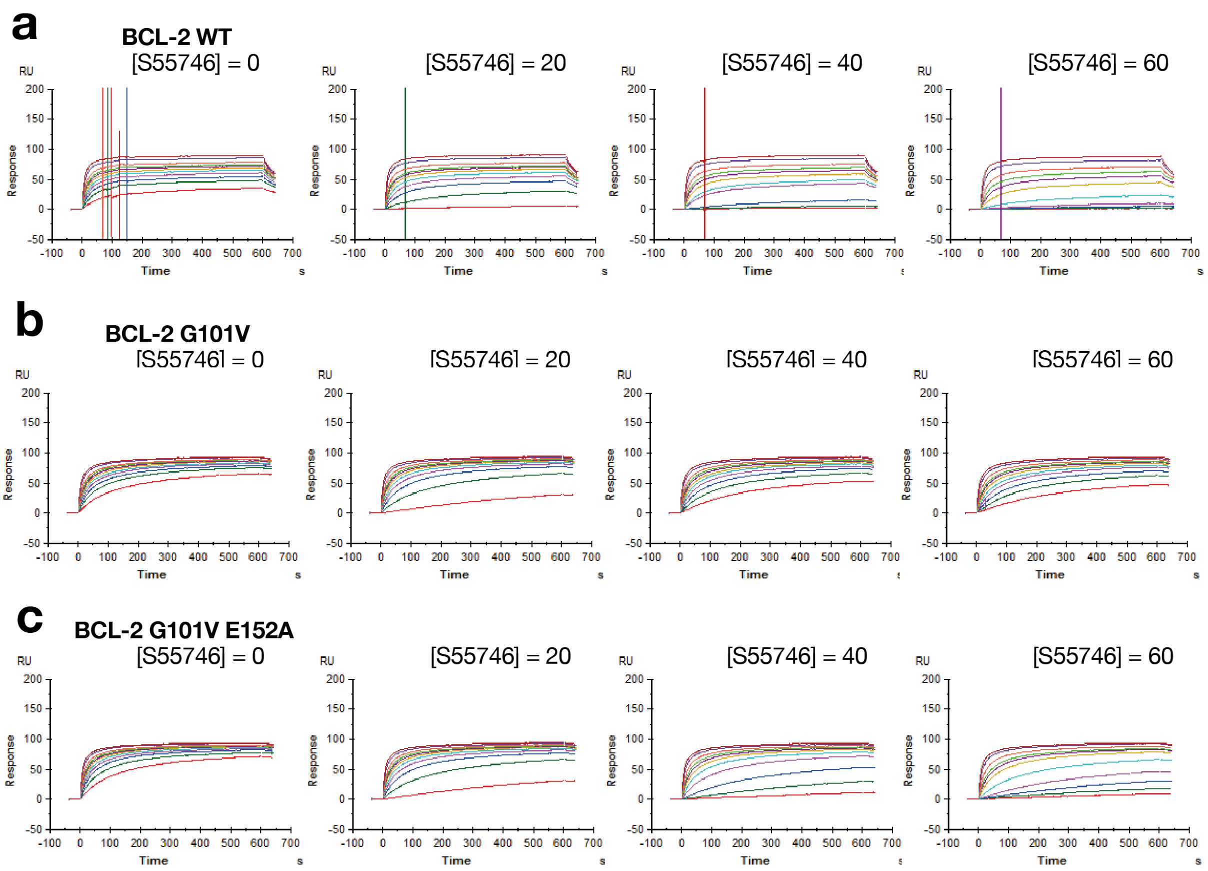


**Supplementary Figure 6. BCL-2 mutants with S55746 steady-state SPR sensograms.** Double referenced sensorgrams for BCL-2 wild-type (WT, **a**), G101V (**b**) and G101V E152A double mutant (**c**) binding to a BIMBH3 peptide immobilised chip in the presence of various S55746 concentrations (0-60 nM). Average response at the end of injection ~600 sec, were plotted as a function of BCL-2 and S55746 concentration in figure 3e-f. Data are representative of at least 2 independent experiments.

**a** BCL-2 WT:Venetoclax

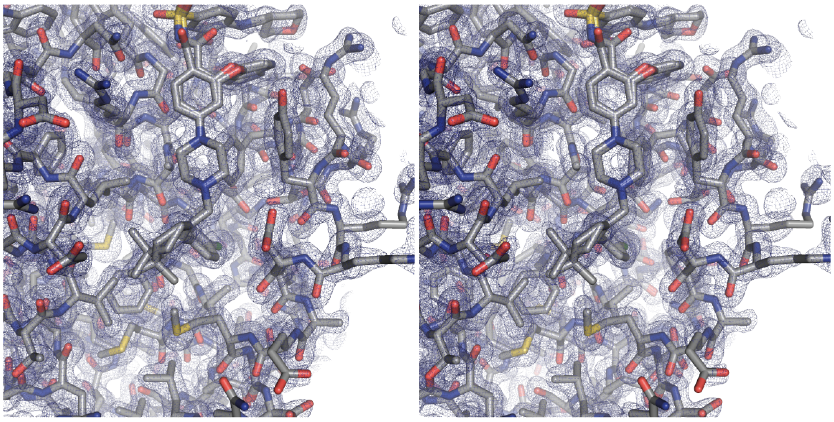

**b** BCL-2 G101V:Venetoclax

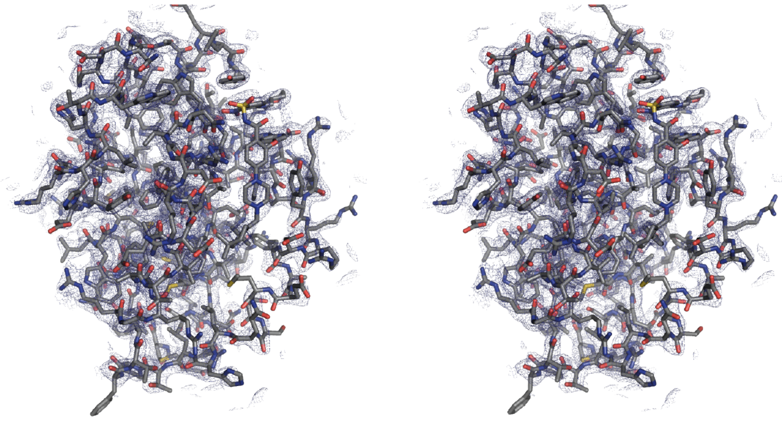

**c** BCL-2 F104L:Venetoclax

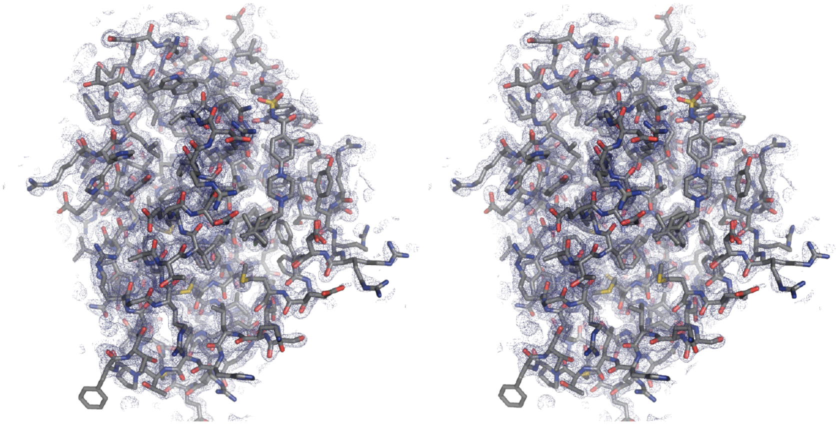

**d** BCL-2 G101V:S55746

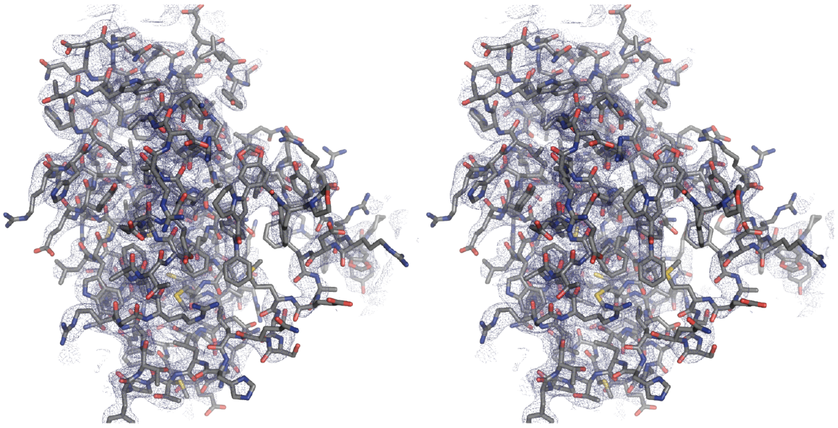

**Supplementary Figure 7. Stereo images of 2Fo-Fc electron density maps contoured at 1 σ for the BCL2 mutant small molecule structures reported.**

| ring position | WT Ven* | WT ABT-263 | WT 4MAN | G101V Ven | G101A Ven |
|---|---|---|---|---|---|
| C1 | 5.06 | 4.91 | 3.88 | 5.39 | 5.01 |
| C2 | 6.08 | 5.43 | 4.09 | 5.75 | 6.03 |
| C3 | 6.22 | 6.01 | 5.04 | 6.17 | 6.17 |
| C4 | 6.43 | 5.85 | 5.70 | 6.41 | 6.38 |
| C5 | 6.22 | 5.05 | 5.52 | 6.20 | 6.17 |
| C6 | 5.61 | 3.98 | 4.67 | 5.52 | 5.56 |
| **RMSD** | **-** | **0.73** | **1.12** | **0.23** | **0.03** |

**Supplementary Table 1. Distance between compounds and BCL-2 residue L137 on helix 4.** Distances are reported in ångström (Å) from each carbon in the 4-4-dimethylcyclohex-1-ene (venetoclax), 5-5-dimethylcyclohex-1-ene (ABT-263), 4′-biphenyl (compound 1) relative to the L137 Cα. *venetoclax showed two conformations for the cyclohex-1-ene ring the A conformer was used as a reference as it matched the conformers from the ABT-263 and G101V:venetoclax structures. See structural analyses for further details.

| **BCL2 variant** | **Ligand** | $k_a$ ($M^{-1}s^{-1}$) | $k_d$ ($s^{-1}$) | $K_D$ (M) | $R_{max}$ (RU) | $\chi^2$ ($RU^2$) |
|---|---|---|---|---|---|---|
| G101A | BIMBH3 | 1.07E+06 | 2.87E-04 | 2.67E-10 | 33 | 0.08 |
| G101A | BIMBH3 | 1.02E+06 | 2.22E-04 | 2.17E-10 | 35 | 0.22 |
| G101A | BIMBH3 | 4.47E+05 | 8.57E-05 | 1.92E-10 | 395 | 75 |
| F104C | BIMBH3 | 5.50E+05 | 4.86E-05 | 8.84E-11 | 102 | 1.1 |
| F104C | BIMBH3 | 2.85E+05 | 4.17E-05 | 1.46E-10 | 248 | 11 |
| F104C | BIMBH3 | 2.96E+05 | 8.38E-05 | 2.83E-10 | 230 | 12 |
| F104C | BAXBH3 | 1.67E+06 | 7.09E-03 | 4.25E-09 | 123 | 4.0 |
| F104C | BAXBH3 | 2.00E+06 | 7.91E-03 | 3.96E-09 | 90 | 3.8 |
| F104C | BAXBH3 | 2.06E+06 | 7.90E-03 | 3.84E-09 | 86 | 3.9 |
| E152A | BIMBH3 | 9.84E+05 | 4.34E-04 | 4.41E-10 | 340 | 17 |
| E152A | BIMBH3 | 7.23E+05 | 2.04E-04 | 2.82E-10 | 89 | 1.1 |
| E152A | BAXBH3 | 2.36E+10 | 9.86E+01 | 4.18E-09 | 29 | 0.8 |
| E152A | BAXBH3 | 4.19E+06 | 1.23E-02 | 2.93E-09 | 71 | 5.0 |
| G101V, E152A | BIMBH3 | 6.89E+05 | 6.24E-04 | 9.05E-10 | 204 | 15 |
| G101V, E152A | BIMBH3 | 6.66E+05 | 2.38E-04 | 3.57E-10 | 87 | 1.1 |
| G101V, E152A | BAXBH3 | 4.56E+09 | 2.60E+01 | 5.70E-09 | 29 | 0.7 |
| G101V, E152A | BAXBH3 | 1.19E+06 | 6.34E-03 | 5.31E-09 | 82 | 2.3 |

**Supplementary Table 2. SPR direct binding fit parameters.** The kinetic parameters for direct binding between BCL-2 variants and either BIMBH3 or BAXBH3 described in Table 2 and Supplementary Figure 4. Data are shown for each independent experiment used in the analysis.

| Primer purpose | Primer sequence (5' to 3') | |
|---|---|---|
| | *Forward* | *Reverse* |
| *Bacterial expression plasmids* | | |
| pGEX sequencing | GGGCTGGCAAGCCACGTTTGGTG | |
| pGEX sequencing | | GAAACGCGCGAGGCAGATCG |
| G101V mutation | CCCTGCGCCAGGCGGTCGATGACTTTAGCCG | CGGCTAAAGTCATCGACCGCCTGGCGCAGGG |
| G101A mutation | CCCTGCGCCAGGCGGCCGATGACTTTAGCCG | CGGCTAAAGTCATCGGCCGCCTGGCGCAGGG |
| F104L mutation | CAGGCGGGCGATGACTTAAGCCGTCGCTATCGTCGC | ACGATAGCGACGGCTTAAGTCATCGCCCGCCTGG |
| F104C mutation | CAGGCGGGCGATGACTGTAGCCGTCGCTATCGTCGC | ACGATAGCGACGGCTACAGTCATCGCCCGCCTGG |
| E152A mutation | GCATTGTCGCATTTTTCGCATTTGGCGGTGTTATGTGCGTC | GACGCACATAACACCGCCAAATGCGAAAAATGCGACAATGC |
| *mamalian expression plasmids* | | |
| G101V mutation reaction 1 | GAATTAGATCTTTCGAAATCACCA | CCTGGCGGAGGGTCAGGTGGA |
| G101V mutation reaction 2 | TGGTCCACCTGACCCTCCGCCAGGCAGTTGACGACTTCTCCCG CCGCTACCGCCGCG | TTATCCTGGATCCAGGTGTGCAGGT |

**Supplementary Table 3. Sequences of oligonucleotide primers used.**
